# Raman spectroscopic analysis of intracellular ice-induced degradation of mesenchymal stromal cells

**DOI:** 10.1016/j.bpj.2026.04.022

**Published:** 2026-04-21

**Authors:** Yuki Uno, Risa Hokkoku, Jun Okuda, Tetsuji Nakamura, Masahiro Kino-oka

**Affiliations:** 1 Department of Biotechnology, Graduate School of Engineering, The University of Osaka, 2-1 Yamadaoka, Suita-shi, Osaka 565-0871, Japan; 2 Research Base for Cell Manufacturability, Graduate School of Engineering, The University of Osaka, 2-1 Yamadaoka, Suita-shi, Osaka 565-0871, Japan; 3 Joint Research Laboratory (Iwatani) for Cell Storage & Transport Technology, Graduate School of Engineering, The University of Osaka, 2-1 Yamadaoka, Suita-shi, Osaka 565-0871, Japan

## Abstract

Cryopreservation enables the long-term maintenance of cellular characteristics, thereby serving as a foundation for establishing a stable supply chain for cells. However, during the freezing process, which is part of cryopreservation, phase-transition-associated biophysical events, such as the formation of intracellular ice crystals (IICs), have the potential to induce cellular degradation. During freezing, the cooling rate influences cellular dehydration and acts as a balancing factor between IIC formation and cellular shrinkage. In particular, IIC formation has been regarded as a lethal event, and various studies have focused on elucidating the underlying mechanisms. However, studies investigating the mechanisms of cellular degradation caused by IIC formation remain limited. The objective of this study is to investigate the degradation mechanisms of mesenchymal stromal cells induced by IIC formation during the freezing process at different cooling rates (1 and 5 K/min). Cell viability assessments at multiple time points after thawing suggested that the 5 K/min group exhibited an increase in the population of cells that lost membrane integrity. To investigate the mechanisms underlying membrane integrity loss, Raman spectroscopic microscopy was employed to analyze changes in cellular size and IIC formation during the freezing process. The analyses suggested that, compared to the 1 K/min group, the 5 K/min group exhibited increased IIC formation and a more uniform spatial distribution, as well as the presence of a specific subpopulation characterized by increased cellular size. These biophysical events may be associated with the loss of membrane integrity after thawing. In this study, cellular degradation during freezing at different cooling rates was analyzed from both biological and physical perspectives. Our findings advance understanding of the mechanisms of cellular degradation induced by IICs and are expected to offer valuable insights for optimizing cell freezing processes.

## Significance

Intracellular ice crystal (IIC) formation has been recognized as a lethal event, and various studies have sought to elucidate its mechanisms. However, studies investigating the mechanisms of cellular degradation caused by IIC formation remain limited. We investigated the degradation mechanisms of mesenchymal stromal cells by combining cell viability assessments at multiple time points after thawing with Raman spectroscopic analysis. This study suggested that faster cooling induced post-thaw membrane integrity loss, increased IIC formation with relatively uniform spatial distribution, and led to the emergence of a specific subpopulation exhibiting an expanded cellular size. Our findings advance understanding of the mechanisms of cellular degradation induced by IICs and are expected to offer valuable insights for optimizing cell freezing processes.

## Introduction

Cryopreservation is a technology that enables the long-term maintenance of cellular characteristics by suppressing or halting intracellular and extracellular reactions.^1^^,^^2^ This technology facilitates the establishment of cell banks and allows for the design of flexible transportation systems.^3^^,^^4^ Consequently, it supports the development of a stable supply chain capable of providing cells with consistent quality. Moreover, it is expected to enhance the reproducibility of outcomes across diverse areas of life sciences, ranging from fundamental research to clinical applications.^5^^,^^6^ However, during the freezing process in cryopreservation, biophysical events involving phase transitions inside and outside the cells can occur, which may lead to cellular degradation.^7^

One of the parameters involved in these events is the cooling rate. In various cell types, it is well known that post-thaw cell viability follows an inverse U-shaped curve as a function of cooling rate.^8^ To interpret the cellular response to cooling rate, Mazur and his colleagues proposed the two-factor hypothesis.^9^ During cooling, cellular dehydration is induced by osmotic pressure gradients across the cell membrane, which are driven by extracellular ice crystal (EIC) formation. At cooling rates faster than the optimal rate yielding the highest cell viability, physical damage from intracellular ice crystal (IIC) formation due to insufficient dehydration becomes dominant. In contrast, at slower cooling rates, chemical damage resulting from solute concentration caused by excessive dehydration predominates. Recent studies have reported that cells may become osmotically inactive below the intracellular glass transition temperature, which markedly suppresses dehydration.^10^^,^^11^ These studies suggest that the cooling rate above the intracellular glass transition temperature may be a major determinant of the extent of dehydration.

Formation of IICs has long been regarded as a lethal event for cells.^12^ Accordingly, various studies have focused on elucidating the mechanisms of IIC formation.^13^^,^^14^^,^^15^^,^^16^^,^^17^^,^^18^ For example, in 1965, Mazur proposed the hypothesis that EICs promote the formation of IICs through pores in the cell membrane.^14^ Meanwhile, in 1990, Muldrew and McGann proposed the hypothesis that osmotic-stress-induced damage to the cell membrane promotes the formation of IICs.^13^ In their study, IICs were detected by observing the optical darkening of cells during freezing, commonly called flashing. Although flashing enables the detection of the presence or absence of IICs, quantifying the extent of IIC formation within individual cells and obtaining detailed information regarding their spatial distribution remains challenging. Recently, a novel approach has been reported that detects IICs as unstained regions by staining intracellular components, such as nuclei with fluorescent dyes and observing them under fluorescence microscopy.^19^ This approach has made it possible to quantitatively measure the number and size of IICs. However, caution should be exercised, as the evaluation was performed in the presence of fluorescent molecules. Although the influence of molecules on biophysical events during freezing remains unclear, label-free evaluation is desirable for capturing the native events.

Raman spectroscopic microscopy (RSM) enables label-free detection of molecular vibrations, which provide information about molecular species.^20^ RSM has been applied in cryobiology to elucidate the mechanisms of action of cryoprotective agents (CPAs)^21^ and cellular degradation induced by temperature fluctuations during storage.^22^ Yu et al. employed RSM to detect IICs and established an index, referred to as *Aic* in their study, to quantify the fraction of the cellular region occupied by the ice crystals.^23^ They used *Aic* to propose the hypothesis that the distance between EICs and the cell membrane is associated with the IIC formation.^16^ Furthermore, their findings indicated that IIC formation does not necessarily represent a lethal event for the cells.^16^ As mentioned above, although multiple studies have investigated the mechanisms of IIC formation, understanding of how IICs contribute to cellular degradation remains limited.

In this study, we investigated the degradation mechanisms of mesenchymal stromal cells (MSCs) during the freezing process at different cooling rates. Based on the post-thaw viability at multiple time points, we retrospectively estimated patterns of cellular degradation. Also, using RSM, we quantitatively evaluated changes in cellular size and IIC formation and analyzed the spatial distribution of IICs. Thus, the degradation mechanisms of MSCs during the freezing process were investigated from both biological and physical perspectives.

## Materials and methods

### Cell preparation

In this study, human bone marrow-derived MSCs (lot number 19TL28098, Lonza Japan, Tokyo, Japan) were used in all experiments. The cells were seeded at a density of 5 × 10^3^ cells/cm^2^ in polystyrene culture plates and cultured for 120 h at 310 K in a humidified atmosphere with 5% CO_2_. A commercially available culture medium (Mesenchymal Stem Cell Growth Medium BulletKit, Lonza Japan) was used for the culture. At 72 h of culture, the entire medium was replaced with fresh medium. At 120 h of culture, the entire medium was removed from the culture plate, and the cells were washed twice with phosphate-buffered saline (PBS; pH 7.4, Thermo Fisher Scientific, MA, USA), then detached from the plate by treatment with trypsin (trypsin-EDTA, phenol red, Thermo Fisher Scientific) for 5 min at 310 K. Fresh medium was added, and the cell suspension was collected into a centrifuge tube and centrifuged at 180 × *g* for 5 min. After centrifugation, the supernatant was removed, and the cell pellet was resuspended in fresh culture medium. Then, the live cell number was measured using an automated cell counter based on the trypan blue exclusion assay (TC20, Bio-Rad Laboratories, CA, USA). Subsequent measurements of live cell number were performed using the same method. The cell suspension was centrifuged at 180 × *g* for 5 min. After centrifugation, the supernatant was removed, and the cell pellet was resuspended in a commercially available cryopreservation solution (STEM-CELL BANKER GMP grade; ZENOGEN PHARMA, Fukushima, Japan) containing 10% dimethyl sulfoxide as a CPA to achieve a final concentration of 1 × 10^6^ cells/mL. The cell suspension was utilized in either post-thaw viability assessments or Raman spectroscopic analysis.

### Post-thaw viability assessments

**Figure 1:**
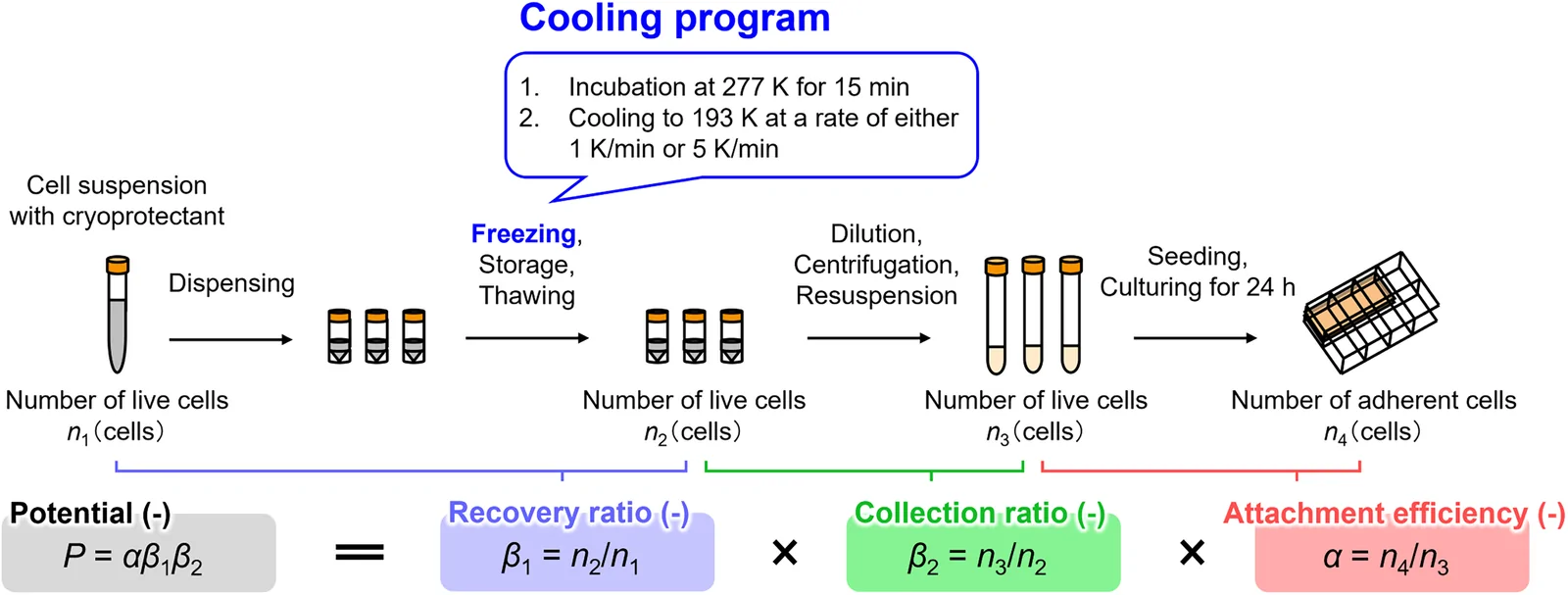
Schematic overview of post-thaw viability assessments. In this study, the number of suspended live cells or adherent cells was measured at multiple time points. Post-thaw viability was assessed using three indicators, *β*_1_, *β*_2,_ and *α*, as well as their product, *P.*

Cell viability was assessed at multiple time points (Figure 1). The live cell number in the cryopreservation solution was measured, and 0.5 mL of the cell suspension was dispensed into each cryotube. To cool the cell suspension, a programmable freezer (Program Deep Freezer FZ-2000; STREX, Osaka, Japan) equipped with a Stirling engine was used, as described in our previous report.^24^^,^^25^ The cryotubes were placed on an aluminum tube holder in the freezer, incubated at 277 K for 15 min, and then cooled to 193 K at a controlled rate of either 1 or 5 K/min. In the freezer, temperature control was based on the temperature of the holder. After cooling, the cryotubes were stored for at least 24 h in the vapor phase of a storage tank with liquid nitrogen.

The measured cooling rate of the cryopreservation solution was 1.50 ± 0.17 K/min in the 1 K/min group and 5.62 ± 0.20 K/min in the 5 K/min group (*n* = 6 cryovials per group). The cooling rate was determined by inserting T-type thermocouples (Teijin Engineering, Osaka, Japan) into a cryovial containing 0.5 mL of the solution and was defined as the rate of temperature decrease from the melting point following ice nucleation to 233 K. Note that the temperature of the solution rises toward its melting point due to the release of latent heat after experiencing supercooling. This creates a temperature gap between the solution and the holder. Consequently, during the subsequent re-cooling process, the cooling rate of the solution becomes faster than the nominal rate.

To assess the post-thaw viability, the stored cryotubes were warmed by immersion in water at 310 K for 2.5 min. After warming, the live cell number was measured for each cryotube. The recovery ratio (*β*_1_), defined as the ratio of the live cell number before freezing to that after thawing, was calculated using Equation 1:

(1) $$\beta_{1\;}=\frac{n_{2}}{n_{1}}\text{,}$$

where *n*_1_ is the number of live cells before freezing and *n*_2_ is the number of live cells after thawing. To remove the CPAs, 0.4 mL of cell suspension from the cryotubes was added to centrifuge tubes with 3 mL of fresh culture medium. Then, the cell suspension was centrifuged at 180 × *g* for 5 min. After removing the supernatant, the cell pellet was resuspended in 0.4 mL of fresh culture medium. For each centrifuge tube, the live cell number was measured. The collection ratio (*β*_2_), defined as the ratio of the number of live cells after thawing to that after removing CPAs, was calculated using Equation 2:

(2) $$\beta_{2\;}=\frac{n_{3}}{n_{2}}\text{,}$$

where *n*_3_ is the number of live cells after removal of CPAs. Based on the results of cell count, live cells were seeded at a density of 2.5 × 10^3^ cells/cm^2^ in an eight-well culture plate at 310 K in a humidified atmosphere containing 5% CO_2_. At 24 h of culture, three images per well were captured using phase-contrast microscopy, and adherent cells were manually counted. Attachment efficiency (*α*), defined as the ratio of the number of seeded live cells to that of adherent cells at 24 h of culture, was calculated using Equation 3:

(3) $$\alpha =\frac{n_{4}}{n_{3}}\text{,}$$

where *n*_4_ is the number of adherent cells at 24 h of culture. The cell potential (*P*), defined as the product of *β*_1_, *β*_2_, and *α*, was calculated using Equation 4:

(4) $$P={\alpha \beta }_{1}\beta_{2}\text{.}$$

### Measurements of Raman spectra

The RSM (alpha300 R, WITec, Ulm, Germany) equipped with a cooling and heating stage (THMS600E, Linkam Scientific Instruments, Salford, UK) was used. On the stage, a cover glass (0.12–0.17 mm × φ22 mm, Matsunami Glass, Osaka, Japan), a spacer (Shim ring, 0.015 × φ16 × φ12 mm, Iijima Seiki, Nagano, Japan), 8 μL of cell suspension, and a cover glass were placed, in that order. A 532 nm excitation laser was irradiated onto the cell suspension on the stage through a 50× objective lens (Objective LD EC, Epiplan-Neofluar, 50×/0.55 differential interference contrast [DIC], NA: 0.55, W.D.: 9.1 mm, Carl Zeiss Microscopy, NY, USA). The Raman scattered light was acquired as a Raman spectrum using an objective lens, a spectrometer (UHTS300, WITec), and a detector (EMCCD camera, Andor Technology, Belfast, Northern Ireland).

The wavenumber ranges used in this study, and the corresponding molecular information, are shown in Table 1. We detected cells by analyzing the amide Ⅰ and alkyl C=C stretching signals arising from proteins and lipids distributed across the cellular structure.^27^ These signals have been employed for the detection of cells after freezing.^16^^,^^21^^,^^23^^,^^28^^,^^29^ In the following section, these two signals are collectively referred to as the amide I signal for simplicity.

**Table 1:** Wavenumber assignments for Raman spectra

Substances	Wavenumber range (cm^−1^)	Assignments
Proteins, lipids	1,610–1,710	amide Ⅰ and C=C stretching^26^
Ice	3,087–3,162	OH stretching^26^

First, the cell suspension was incubated at 277 K for 5 min, after which bright-field images and Raman spectra were acquired. Subsequently, the suspension was cooled to 193 K at rates of 1 or 5 K/min, followed by incubation for 5 min. After capturing bright-field images, the laser was irradiated over a 40 μm range, including a cell along the *z* axis to obtain Raman spectra. The amide I signal intensity, represented as a function of height along the *z* axis, was fitted using the Lorentzian function, and the height corresponding to the maximum signal intensity was determined. At the determined height, Raman spectra were again acquired.

To minimize cellular degradation caused by measurement, the laser irradiation time per 1 μm^2^ was set to 0.6 s/μm^2^, and the laser intensity was set to 40.0 mW, confirming that no visible morphological changes of cells occurred following eight measurements. Note that the actual laser intensity measured with a light power meter (PM100D, Thorlabs, NJ, USA) through the objective lens and cover glass was 26.5 mW.

### Analysis of Raman spectra

Raman spectra were analyzed using dedicated software (WITec Project Data Evaluation Software, WITec) to quantitatively evaluate changes in cellular size and IIC formation and to qualitatively assess the spatial distribution of IICs.

**Figure 2:**
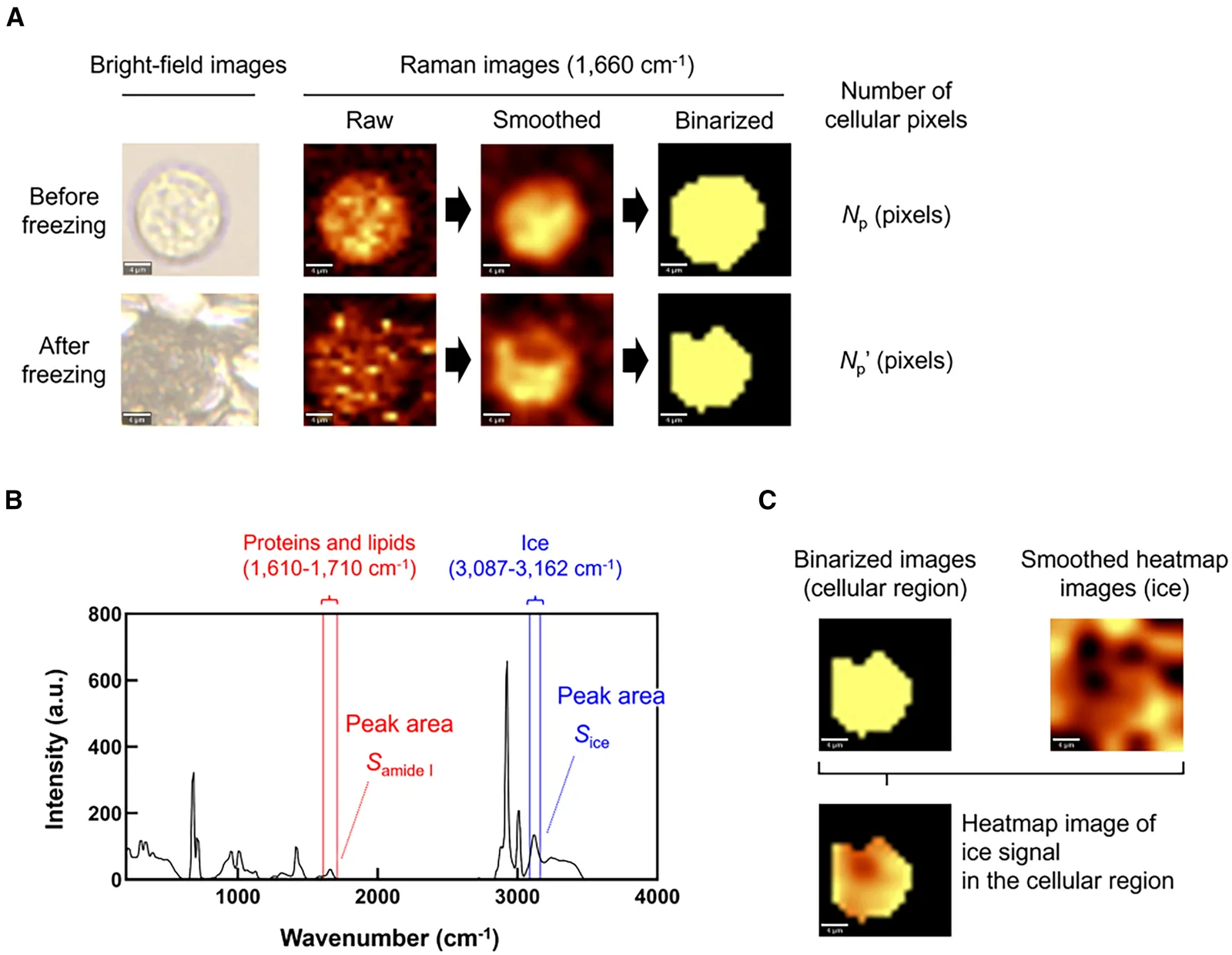
Overview of Raman spectroscopic analysis. (A) Detection process of the cellular region before and after freezing. The number of cellular pixels was counted from the binarized Raman image at 1,660 cm^−1^. (B) Representative baseline-corrected average Raman spectrum of the cellular region after freezing. Peak areas were calculated in the wavenumber ranges of 1,610–1,710 cm^−1^ for proteins and lipids and 3,087–3,162 cm^−1^ for ice. (C) Generation of an ice signal heatmap within the cellular region. The binarized Raman image of the cellular region and the smoothed Raman heatmap image of ice signals were used.

Changes in cellular size were quantitatively analyzed using the following procedure (Figure 2A). To reduce spectrum noise, a nine-point moving-average filter was applied to the Raman spectra at each pixel within the region of interest (ROI) after removing the effects of cosmic rays. To perform image smoothing, a 5 × 5-pixel kernel was applied to the Raman image at each pixel within the ROI. Baseline correction was applied to the average Raman spectrum of all pixels within the ROI using rolling-ball algorithms. Pixels were classified as cellular or noncellular based on their signal/noise ratios. Noise level was estimated from the silent spectral range at 1,900–2,000 cm^−1^ as the difference between the maximum and minimum peak intensity. Pixels were classified as cellular if the signal intensity at 1,660 cm^−1^ was at least five times greater than the noise level, although those in clearly cell-free regions based on bright-field images were re-classified as noncellular pixels. Furthermore, under the assumption that the cellular cross-section is simply connected, noncellular pixels enclosed by cellular pixels were re-classified as cellular pixels. The number of cellular pixels before freezing was defined as *N*_p_ (pixels) and that after freezing as *N*_p_ʹ (pixels). The ratio *N*_p_ʹ/*N*_p_ (−) was used as an index to evaluate the changes in cellular size. Hereinafter, regions composed of cellular pixels are referred to as cellular regions.

IIC formation was quantitatively analyzed using the following procedure (Figure 2B). The average Raman spectrum of cellular regions after freezing, which underwent the same smoothing process as described above, was calculated. The peak area was defined as the integral of the Raman spectrum within a specific wavenumber range, bounded by a baseline drawn as a straight line connecting the start and end points of the spectrum in that range. The peak areas from 1,610 to 1,710 cm^−1^ and from 3,087 to 3,162 cm^−1^ were designated *S*_amideⅠ_ (−) and *S*_ice_ (−), respectively. The ratio *S*_ice_/*S*_amideⅠ_ (−) was used as an index to evaluate IIC formation.

The procedure for analysis of the spatial distribution of IICs is described below (Figure 2C). The signal intensity for each pixel was integrated using the binarized Raman image of the cellular region and the smoothed Raman heatmap image of ice signals, thereby generating a Raman heatmap image of ice signals within the cellular region.

### Statistical analysis

**Figure 3:**
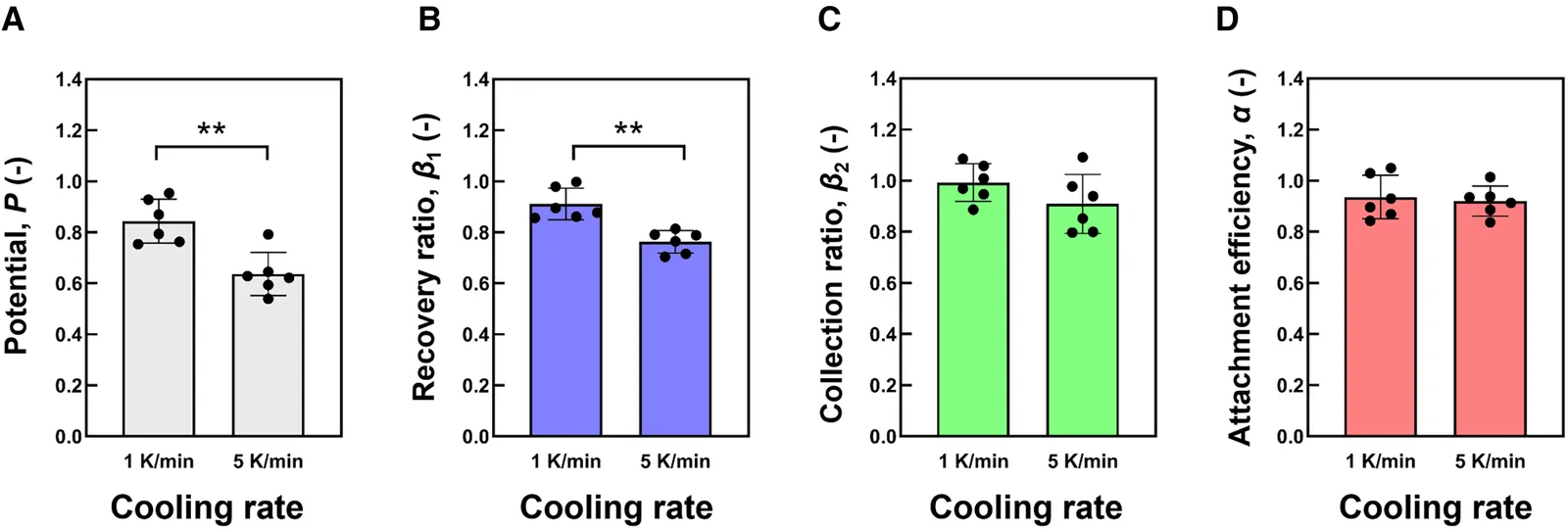
Results of post-thaw viability evaluation under different cooling conditions. Each group shows the results of (A) *P*, (B) *β*_1_, (C) *β*_2_, and (D) *α*, respectively. The graphs display mean values with standard deviation error bars and individual data points. Each graph is based on six data points, obtained from three cryotubes per experiment across two independent experiments. Statistical analysis was performed between the two groups using an unpaired two-tailed *t* test: ^∗∗^*p* < 0.01. Any comparison with a *p*-value greater than 0.05 was determined to have no statistically significant difference.

**Figure 4:**
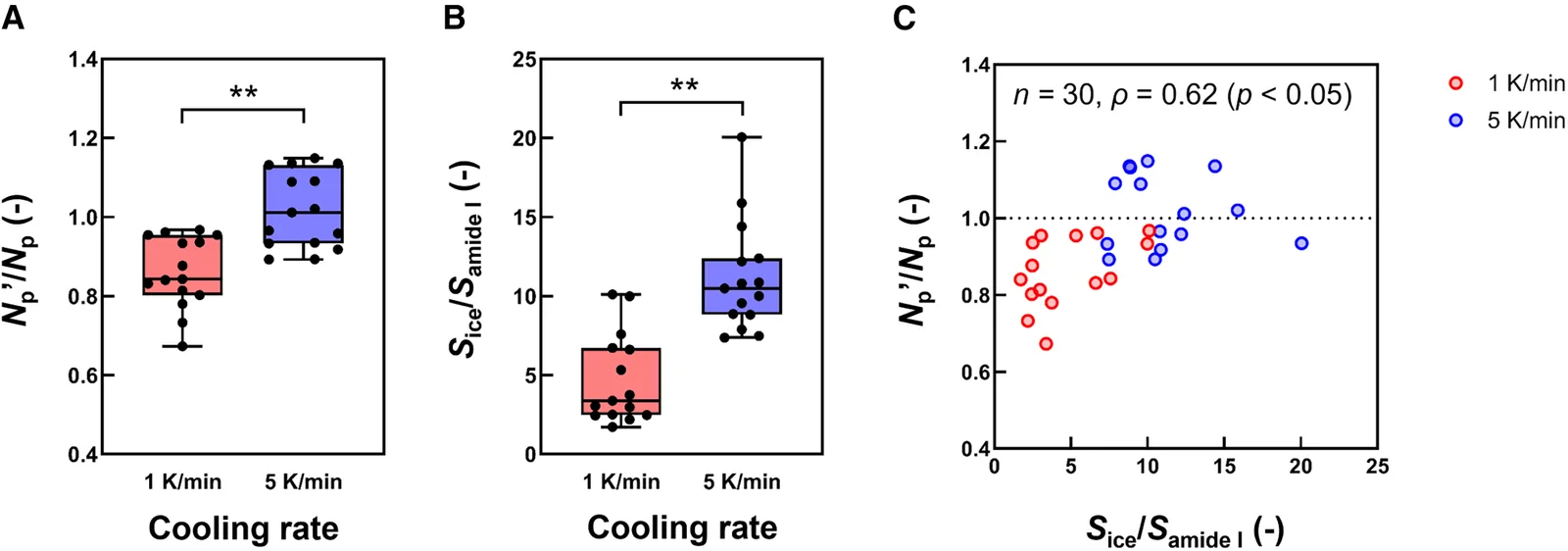
Quantitative Raman spectroscopic analysis of biophysical events during freezing (A and B) Results of quantitative evaluation of (A) changes in cellular size, *N*_p_ʹ/*N*_p,_ and (B) IIC formation, *S*_ice_/*S*_amideⅠ_, under different cooling conditions. The whiskers represent the minimum and maximum values. The bottom and top of the box indicate the first quartile and third quartile, respectively, and the line inside the box represents the median. These graphs were based on 15 data points, obtained from five cells per experiment across three independent experiments. (C) 2D scattering plot of *N*_p_ʹ/*N*_p_ and *S*_ice_/*S*_amideⅠ_. The Spearman’s rank correlation coefficient, *ρ*, is indicated in the graph. Statistical analysis was performed between the two groups using the Mann-Whitney U test for *N*_p_ʹ/*N*_p_ and *S*_ice_/*S*_amideⅠ_: ^∗∗^*p* < 0.01. To evaluate the significance of the correlation coefficient, a test for zero correlation was conducted, and the *p*-value is indicated on the graph.

Statistical analysis was conducted using commercially available scientific 2D graphing and statistics software (Prism v.10, GraphPad, CA, USA). In Figure 3, all pairwise comparisons were performed using unpaired two-tailed *t* tests. In Figures 4A and 4B, Mann-Whitney U tests were performed for pairwise comparisons, while a test for no correlation was performed in Figure 4C. In all cases, a significant level of 5% (*p*-value = 0.05) was applied. Accordingly, any comparison yielding a *p*-value of 0.05 or higher was determined to be statistically nonsignificant. Detailed descriptions of the statistical analyses are provided in the captions of each figure.

## Results

### Post-thaw viability assessments

The value of *P* exhibited a statistically significant difference between the 1 and 5 K/min groups, with the 5 K/min group showing a lower mean value (Figure 3A). To analyze the cause of the lower mean value of *P* observed in the 5 K/min group, *P* was decomposed into three evaluation indices: *β*_1_, *β*_2_, and *α*. The value of *β*_1_ exhibited a statistically significant difference between the 1 and 5 K/min groups, with the 5 K/min group showing a lower mean value (Figure 3B). In contrast, no statistically significant differences were observed in *β*_2_ and *α* between the 1 and 5 K/min groups (Figures 3C and 3D). Based on the results, compared to the 1 K/min group, the lower value of *P* observed in the 5 K/min group was found to be attributable to the lower value of *β*_1_.

### Quantitative analysis of changes in cellular size and IIC formation

The value of *N*_p_ʹ/*N*_p_ exhibited a statistically significant difference between the 1 and 5 K/min groups, with the 5 K/min group tending to show relatively higher values (Figure 4A). In the 1 K/min group, the value of *N*_p_ʹ/*N*_p_ was below unity in all 15 cells, whereas in the 5 K/min group, it was below unity in seven cells and above unity in the remaining eight cells. The value of *S*_ice_/*S*_amideⅠ_ exhibited a statistically significant difference between the 1 and 5 K/min groups, with the 1 K/min group tending to show relatively lower values (Figure 4B). In the 2D scattering plot of *N*_p_ʹ/*N*_p_ and *S*_ice_/*S*_amideⅠ_, color coding based on both cooling rate conditions revealed two visually separable clusters with partial overlapping (Figure 4C). Spearman’s rank correlation coefficient (*ρ*) was calculated between these indices across all 30 cells, revealing a significant positive correlation (*ρ* = 0.62, *p* < 0.05).

### Spatial distribution of IICs

**Figure 5:**
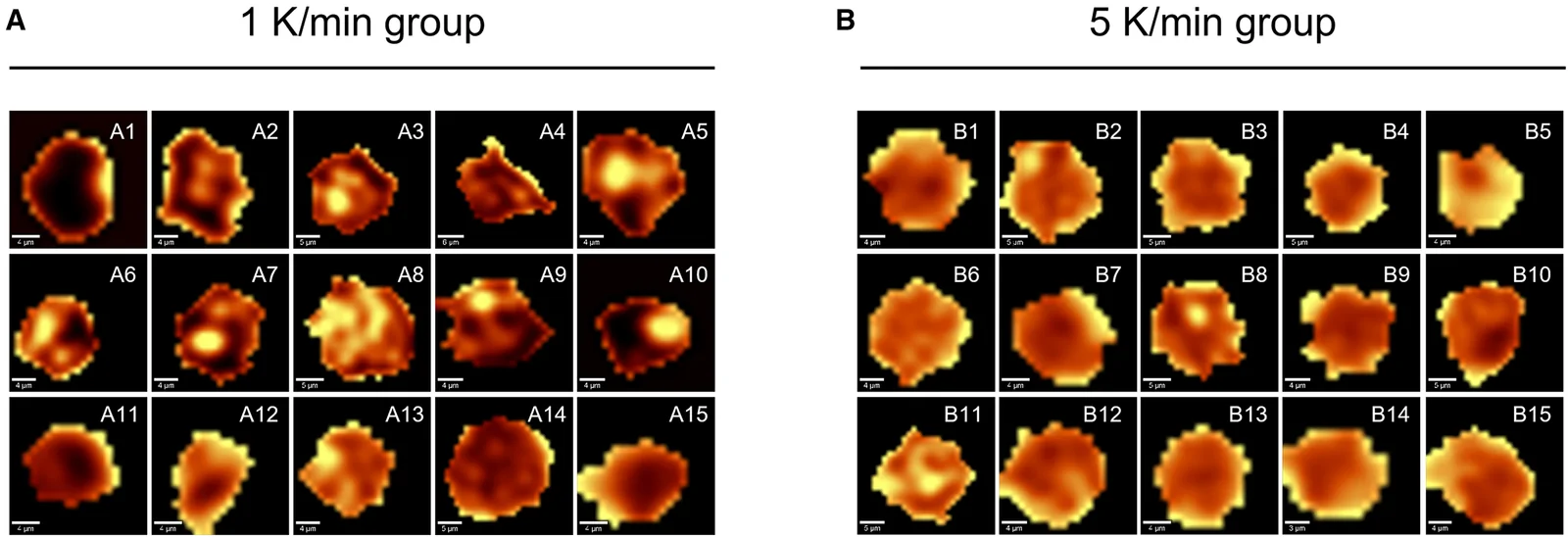
Qualitative Raman spectroscopic analysis of biophysical events during freezing Raman heatmap of ice signal intensity in the cellular region in the (A) 1 and (B) 5 K/min groups. A scale bar is provided in the bottom left corner, and an identification number is shown in the top right corner of each image. A1–A5, A6–A10, A11–A15, B1–B5, B6–B10, and B11–B15 were acquired from six independent experiments, with five cells scanned per experiment using Raman spectroscopic microscopy.

Raman heatmaps were generated to visualize the ice signal intensity within the cellular region (Figure 5). In the 1 K/min group, a biased distribution pattern of the ice signal, represented by A1, A2, A7, and A10, was identified in multiple cells. Meanwhile, in the 5 K/min group, compared with the 1 K/min group, the ice signal was observed to be more uniformly distributed throughout the entire cellular region. In both groups, the ice signal in the peripheral region of the cellular region was a shared characteristic.

## Discussion

The pattern of cellular degradation during freezing at different cooling rates was retrospectively analyzed based on post-thaw viability at multiple time points. Previously, we developed the indices *α* and *β* as post-thaw viability assessment indices for analyzing cellular degradation^30^ and applied them in several studies for the process design of cryopreservation.^22^^,^^25^^,^^31^^,^^32^^,^^33^ Here, *β* corresponds to the product of *β*_1_ and *β*_2_ in this study.

Of the two indices, *α* was defined as the ratio of the number of seeded live cells and adherent cells at 24 h of culture. A decrease in *α* can be interpreted as an increase in the proportion of cell populations in which apoptosis was triggered and/or in which adhesive ability was reduced during cryopreservation.^22^^,^^31^ Conversely, *β* was defined as the ratio of the number of live cells before freezing and after post-thaw CPA removal. When the number of live cells was determined based on the trypan blue exclusion assay, which stains cells that lost membrane integrity,^34^ the decrease in *β* can be interpreted as an increase in the proportion of cell populations in which membrane integrity was lost. However, the CPA removal process involves factors that may affect membrane integrity, such as osmotic stress caused by dilution and mechanical stress caused by centrifugation.^35^^,^^36^ Therefore, a challenge was to distinguish whether the loss of membrane integrity occurred prior to thawing or during the CPA removal process following thawing. In our previous studies, since our analyses primarily focused on the decrease in *α*, this issue did not pose a significant problem.

In this study, *β* was divided into *β*_1_ and *β*_2_ to enable a more detailed analysis of cellular degradation during the freezing process. Compared to the 1 K/min group, only *β*_1_ showed a significant difference in the 5 K/min group, with a lower mean value (Figure 3B). These results suggest that, compared to the 1 K/min group, the 5 K/min group exhibited an increased population of cells whose membranes were damaged during freezing, resulting in a loss of membrane integrity either during freezing or upon thawing. Meanwhile, the absence of a significant difference in *β*_2_ suggests that the tested cooling rate had a comparable impact on tolerance to mechanical stress during CPA removal (Figure 3C). Furthermore, the absence of a significant difference in *α* suggests that the impact of the tested cooling rate on the levels of apoptosis induction and the reduction in adhesion ability was comparable (Figure 3D). According to the two-factor hypothesis, the decrease in cell viability at faster cooling rates is primarily attributed to IIC formation.^7^^,^^9^ Therefore, the increase in the population of cells exhibiting membrane damage in the 5 K/min group may be attributed to biophysical events associated with IIC formation.

To understand the events, we developed quantitative analytical indices: *N*_p_ʹ/*N*_p_ for changes in cellular size and *S*_ice_/*S*_amideⅠ_ for IIC formation, using RSM, inspired by the two-factor hypothesis. The index representing changes in cellular size was defined as the ratio of the pixel number in the cellular region before and after freezing. In several studies, the ratio of the pixel number in the region defined using RSM has been employed as an index for understanding biophysical events.^16^^,^^23^^,^^37^ However, to the best of our knowledge, no previous studies have utilized the ratio of pixel number in the cellular region before and after freezing. Conversely, Yu et al. developed an analytical index for IIC formation, designated *Aic*, which represents the ratio of the pixel number between the cellular and IIC regions,^23^ which has been applied to several studies.^16^^,^^21^^,^^28^ They binarized the Raman heatmap image based on the presence or absence of the OH stretching peak of ice in the Raman spectra to define the IIC region.^23^ This index is suitable for evaluating the fraction of the cellular region occupied by ice crystals. However, binarization results in the loss of information regarding signal intensity. Signal intensity contains information such as molecular density and other relevant factors.^38^ Therefore, *Aic* was not employed for the quantitative analysis of IICs in this study.

In the 1 K/min group, the value of *N*_p_ʹ/*N*_p_ was less than unity in all observed cells and was significantly lower than that in the 5 K/min group (Figure 4A). These results suggest that, in the 1 K/min group, all observed cells experienced shrinkage during freezing, with greater shrinkage than in the 5 K/min group. As previously mentioned, the degree of cellular dehydration during freezing depends on the cooling rate, with faster cooling resulting in a lower degree. Therefore, the cellular shrinkage observed in this study is likely attributable to cellular dehydration. Interestingly, in the 5 K/min group, the value of *N*_p_ʹ/*N*_p_ was below unity in seven cells and above unity in eight cells. These findings suggest the presence of two distinct cell populations in the 5 K/min group: one that shrinks and one that expands during freezing. In the 5 K/min group, the value of *S*_ice_/*S*_amideⅠ_ showed a significantly higher value compared to the 1 K/min group (Figure 4B). Additionally, a positive correlation was observed between the value of *N*_p_ʹ/*N*_p_ and *S*_ice_/*S*_amideⅠ_ (Figure 4C). These results suggest a potential association between increased IIC formation and cellular expansion observed in the 5 K/min group. There are few studies that have reported cellular expansion during freezing, as most existing research has been limited to the tissue.^39^^,^^40^ The expansion observed in the tissue is primarily attributed to the approximately 9% volumetric increase associated with the phase transition of liquid water to ice within it. Given the greater extent of IIC formation in the 5 K/min group, it is possible that the observed cellular expansion is also attributable to the volumetric increase associated with the phase transition. We hypothesized that in the 5 K/min group, the increase in cellular size associated with the phase transition outweighed the decrease in size associated with dehydration in a specific subpopulation, ultimately resulting in the observed cellular expansion. However, as there is currently no direct experimental evidence to support these considerations about the dynamic changes in cellular size, further investigation is required to validate this hypothesis.

During cooling, the cell membrane is known to undergo a phase transition from a liquid-crystalline to a gel state across a wide range of species, including sperm, cells, and bacteria.^10^^,^^41^^,^^42^ Gelation of the membrane induces a rigid and fragile state, potentially rendering the membrane more susceptible to damage.^43^^,^^44^ In this study, direct analysis of the state of the membrane, which has a thickness on the nanometer scale, was not possible due to current technical limitations. In the future, employing high-resolution imaging technologies and/or molecular dynamics simulations to understand membrane fragility may contribute to clarifying the relationship between the biophysical events during freezing and the loss of membrane integrity after thawing.

Additionally, this study demonstrated that the spatial distribution of ice signals varies with the cooling rate (Figure 5). These results suggest that the distribution of IICs varies depending on the cooling rate. In the 1 K/min group, IICs displayed a spatially biased distribution across multiple cells, while in the 5 K/min group, most cells exhibited a relatively uniform distribution of IICs.

Meanwhile, the presence of ice signals at the periphery of the cellular region was a common feature observed under both cooling rate conditions. These results suggest that IICs are present around the cell membrane regardless of the cooling rate tested. The formation of IICs around the cell membrane is consistent with the findings reported by Yu et al.^16^ Because the occurrence of IICs near the cell membrane was common to both cooling rates, their presence alone is likely to exert only a minor effect on cellular degradation.

**Figure 6:**
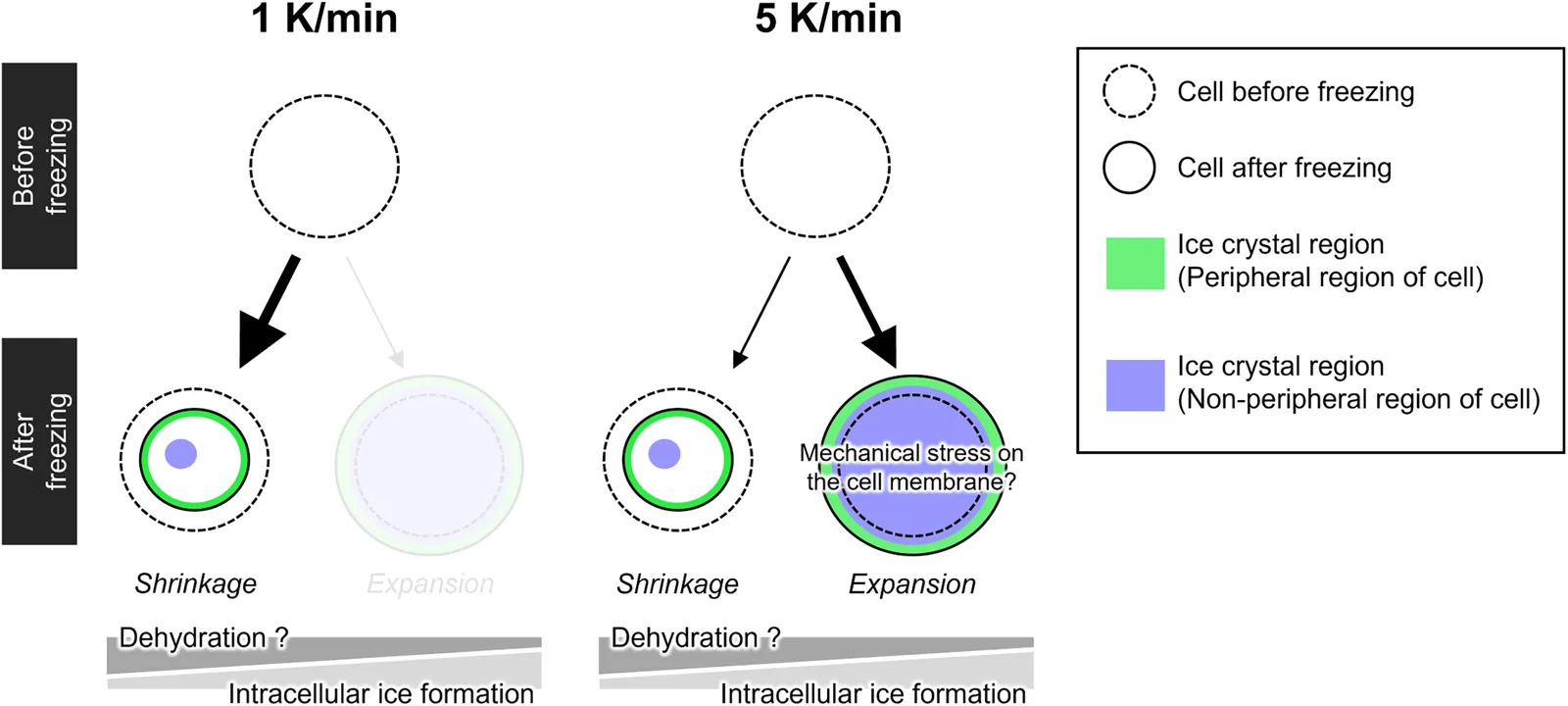
Schematic illustration of the hypothetical biophysical events underlying the degradation of mesenchymal stromal cells during freezing.

Based on these findings, we propose a hypothesis regarding the degradation mechanisms of MSCs occurring under the 1 and 5 K/min cooling conditions (Figure 6). In the present study, Raman spectroscopic analysis suggested that, compared to the 1 K/min group, the 5 K/min group exhibited an increase in IIC formation and its relatively uniform spatial distribution, as well as the presence of a specific subpopulation with expanded cellular size. These biophysical events may exert mechanical stress on the cell membrane. However, further investigation is required to establish the direct causal relationship between these biophysical events during freezing and the loss of membrane integrity after thawing. Furthermore, although post-thaw viability assessments and Raman spectroscopic analyses were conducted under the same cooling conditions in this study, it should be noted that the underlying biophysical events may not be perfectly identical due to factors specific to each experimental setup, such as differences in heat transfer resulting from sample volumes and materials.

## Conclusion

In this study, the degradation of MSCs during freezing processes with different cooling rates was analyzed from both biological and physical perspectives. Post-thaw viability assessments at multiple time points are useful for retrospective analysis of cellular degradation patterns. Furthermore, RSM enables detailed analysis of the mechanisms underlying cellular degradation by visualizing the biophysical events during freezing, which have traditionally been regarded as a black box. Our findings suggest that under the 5 K/min cooling condition, there is an increased population of cells exhibiting loss of membrane integrity after thawing. Moreover, Raman spectroscopic analysis suggests that the 5 K/min cooling promoted greater, uniformly distributed IIC formation, as well as the presence of a specific subpopulation with increased cellular size. These biophysical events are potentially associated with post-thaw loss of membrane integrity. This hypothesis is expected to provide a basis for the design of cryoprotectants for MSCs. Additionally, recent research has increasingly focused on the computer-aided design of cell freezing processes using simulation-based approaches.^25^^,^^33^^,^^45^^,^^46^^,^^47^ The establishment of quantitative indices for analyzing changes in cellular size and IIC formation is expected to facilitate the development of simulation models, thereby improving predictive accuracy and enabling more rigorous validation of model outcomes.

## Data availability

Data from this study will be made available upon reasonable request.

## Acknowledgments

This work was supported by the 10.13039/100009619Japan Agency for Medical Research and Development under grant numbers JP20be0704001 and JP24bk0304004. Much of this work forms the basis of Y.U.’s PhD dissertation.

## Author contributions

Y.U. designed the research, performed the research, contributed analytic tools, analyzed the data, and wrote the manuscript. R.H. designed the research, performed the research, contributed analytic tools, analyzed the data, and wrote the manuscript. J.O. designed the research, performed the research, contributed analytic tools, analyzed the data, and wrote the manuscript. T.N. designed the research and contributed analytic tools. M.K. designed the research and wrote the manuscript.

## Declaration of interests

R.H., J.O., and T.N. are employed by Iwatani Corporation. M.K. received grants for collaborative research from the Iwatani Corporation.

## References

[1] Uno Y., Hayashi Y., Kino-oka M. (2026). Strategies to Enhance Stability of Cryopreservation Processes for Cell-Based Products. Biotechnol. Adv..

[2] Uno Y., Nakamura K., Kino-oka M. (2025). Basic Points to Consider for Cell Storage under the Act on the Safety of Regenerative Medicine in Japan. Regen. Ther..

[3] Chang Y.H., Fujimori Y., Mochiki K. (2025). Practice of Cryopreservation of Cellular Starting Materials from the Asia-Pacific Region: An Industrial Perspective. Ther. Innov. Regul. Sci..

[4] Tyagarajan S., Schmitt D., Rutjens E. (2019). Autologous cryopreserved leukapheresis cellular material for chimeric antigen receptor–T cell manufacture. Cytotherapy.

[5] Selfa Aspiroz L., Mennecozzi M., Whelan M. (2025). Promoting the adoption of best practices and standards to enhance quality and reproducibility of stem cell research. Stem Cell Rep..

[6] Mah N., Kurtz A., Mueller S.C. (2023). The Management of Data for the Banking, Qualification, and Distribution of Induced Pluripotent Stem Cells: Lessons Learned from the European Bank for Induced Pluripotent Stem Cells. Cells.

[7] Gao D., Critser J.K. (2000). Mechanisms of Cryoinjury in Living Cells. ILAR J..

[8] Woods E.J., Thirumala S., Mathew A.J. (2016). Off the shelf cellular therapeutics: Factors to consider during cryopreservation and storage of human cells for clinical use. Cytotherapy.

[9] Mazur P., Leibo S.P., Chu E.H. (1972). A two-factor hypothesis of freezing injury. Evidence from Chinese hamster tissue-culture cells. Exp. Cell Res..

[10] Meneghel J., Kilbride P., Fonseca F. (2019). Physical events occurring during the cryopreservation of immortalized human T cells. PLoS One.

[11] Kilbride P., Meneghel J., Morris J. (2021). The transfer temperature from slow cooling to cryogenic storage is critical for optimal recovery of cryopreserved mammalian cells. PLoS One.

[12] Mazur P., Pinn I.L., Kleinhans F.W. (2007). Intracellular ice formation in mouse oocytes subjected to interrupted rapid cooling. Cryobiology.

[13] Muldrew K., Mcgann L.E. (1990). Mechanisms of intracellular ice formation. Biophys. J..

[14] Mazur P. (1965). The role of cell membranes in the freezing of yeast and other single cells. Ann. N. Y. Acad. Sci..

[15] ASAHINA É. (1962). Frost Injury in Living Cells. Nature.

[16] Yu G., Yap Y.R., Hubel A. (2017). Characterizing Intracellular Ice Formation of Lymphoblasts Using Low-Temperature Raman Spectroscopy. Biophys. J..

[17] Ninagawa T., Eguchi A., Narumi A. (2016). A study on ice crystal formation behavior at intracellular freezing of plant cells using a high-speed camera. Cryobiology.

[18] Higgins A.Z., Karlsson J.O.M. (2013). Effects of intercellular junction protein expression on intracellular ice formation in mouse insulinoma cells. Biophys. J..

[19] Poisson J.S., Acker J.P., Ben R.N. (2019). Modulating Intracellular Ice Growth with Cell-Permeating Small-Molecule Ice Recrystallization Inhibitors. Langmuir.

[20] Palonpon A.F., Ando J., Fujita K. (2013). Raman and SERS microscopy for molecular imaging of live cells. Nat. Protoc..

[21] Yu G., Li R., Hubel A. (2019). Interfacial Interactions of Sucrose during Cryopreservation Detected by Raman Spectroscopy. Langmuir.

[22] Okuda J., Watanabe N., Kino-oka M. (2024). The impact of repeated temperature cycling on cryopreserved human iPSC viability stems from cytochrome redox state changes. Front. Bioeng. Biotechnol..

[23] Yu G., Li R., Hubel A. (2021). Raman Cryomicroscopic Imaging and Sample Holder for Spectroscopic Subzero Temperature Measurements. Methods Mol. Biol..

[24] Hayashi Y., Horiguchi I., Sugiyama H. (2021). Model-based assessment of temperature profiles in slow freezing for human induced pluripotent stem cells. Comput. Chem. Eng..

[25] Hayashi Y., Uno Y., Sugiyama H. (2024). Computer-aided exploration of multiobjective optimal temperature profiles in slow freezing for human induced pluripotent stem cells. Cryobiology.

[26] Li R., Hornberger K., Hubel A. (2020). Cryopreservation of Human iPS Cell Aggregates in a DMSO-Free Solution—An Optimization and Comparative Study. Front. Bioeng. Biotechnol..

[27] Redolfi-Bristol D., Yamamoto K., Pezzotti G. (2025). Mapping Selenium Nanoparticles Distribution Inside Cells through Confocal Raman Microspectroscopy. ACS Appl. Mater. Interfaces.

[28] Louwagie T., Wagner M., Hubel A. (2023). Characterizing cellular membrane partitioning of DMSO using low-temperature Raman spectroscopy. Front. Mol. Biosci..

[29] Pi C.H., Yu G., Hubel A. (2018). Characterizing the “sweet spot” for the preservation of a T-cell line using osmolytes. Sci. Rep..

[30] Kagihiro M., Fukumori K., Kino-oka M. (2018). Kinetic analysis of cell decay during the filling process: Application to lot size determination in manufacturing systems for human induced pluripotent and mesenchymal stem cells. Biochem. Eng. J..

[31] Kagihiro M., Fukumori K., Kino-oka M. (2020). Suppression of time-dependent decay by controlling the redox balance of human induced pluripotent stem cells suspended in a cryopreservation solution. Biochem. Eng. J..

[32] Nair A., Horiguchi I., Kino-oka M. (2022). Development of instability analysis for the filling process of human-induced pluripotent stem cell products. Biochem. Eng. J..

[33] Tamaki R., Hayashi Y., Sugiyama H. (2025). A circular exploration of cryoprotective agents for stem cells using computer-aided molecular design approaches. Digit. Chem. Eng..

[34] Strober W. (2015). Trypan blue exclusion test of cell viability. Curr. Protoc. Immunol..

[35] Fleming Glass K.K., Longmire E.K., Hubel A. (2008). Optimization of a microfluidic device for diffusion-based extraction of DMSO from a cell suspension. Int. J. Heat Mass Transf..

[36] Marquez-Curtis L.A., Dai X.Q., Elliott J.A.W. (2022). Cryopreservation and post-thaw characterization of dissociated human islet cells. PLoS One.

[37] Borek-Dorosz A., Pieczara A., Majzner K. (2024). Raman microscopy reveals how cell inflammation activates glucose and lipid metabolism. Biochim. Biophys. Acta. Mol. Cell Res..

[38] Durickovic I., Stauffer M.T. (2016). Applications of Molecular Spectroscopy to Current Research in the Chemical and Biological Sciences.

[39] Teo K.Y., Dutton J.C., Han B. (2010). Spatiotemporal measurement of freezing-induced deformation of engineered tissues. J. Biomech. Eng..

[40] Seawright A., Ozcelikkale A., Han B. (2013). Role of Cells in Freezing-Induced Cell-Fluid-Matrix Interactions Within Engineered Tissues. J. Biomech. Eng..

[41] Sieme H., Oldenhof H., Wolkers W.F. (2015). Sperm Membrane Behaviour during Cooling and Cryopreservation. Reprod. Domest. Anim..

[42] Gautier J., Passot S., Fonseca F. (2013). A low membrane lipid phase transition temperature is associated with a high cryotolerance of *Lactobacillus delbrueckii* subspecies *bulgaricus* CFL1. J. Dairy Sci..

[43] Castro L.S., Hamilton T.R.S., Assumpção M.E.O.A. (2016). Sperm cryodamage occurs after rapid freezing phase: Flow cytometry approach and antioxidant enzymes activity at different stages of cryopreservation. J. Anim. Sci. Biotechnol..

[44] Raju R., Bryant S.J., Bryant G. (2021). The need for novel cryoprotectants and cryopreservation protocols: Insights into the importance of biophysical investigation and cell permeability. Biochim. Biophys. Acta. Gen. Subj..

[45] Yuan P., Dong X., Gong M. (2025). Modeling and typical cases analyze at the cell-scale of transmembrane transport and intracellular crystallization and recrystallization during the freeze-thaw process. Cryobiology.

[46] Hayashi Y., Kino-oka M., Sugiyama H. (2022). Hybrid-model-based design of fill-freeze-thaw processes for human induced pluripotent stem cells considering productivity and quality. Comput. Chem. Eng..

[47] Scholz B.X., Hayashi Y., Sugiyama H. (2024). Computational fluid dynamics model-based design of continuous forced convection freezing processes for human induced pluripotent stem cells considering supercooling of extracellular solutions. Chem. Eng. Res. Des..

